# Supplementary material for: Contrasting phytoplankton-zooplankton distributions observed through autonomous platforms, in-situ optical sensors and discrete sampling
Source: PLoS One. 2022 Sep 6;17(9):e0273874. doi: 10.1371/journal.pone.0273874 (PMC9447933; doi:10.1371/journal.pone.0273874)
Supplement: S1 Table — Acquisition protocols (A, B and C) used with different combinations of LED color (blue, green and red) and respective flux (Eflux, photons nm-2 100 μs-1), number of acquisitions collected (n) and average (μ) and standard deviation (±) for the optimum combination of intensity and color of the LED that fully saturates the reaction centers during the first flashlet (RσPII). (PDF) [file pone.0273874.s001.pdf]

S1 Table. FRRf acquisition protocols. Acquisition protocols (A, B and C) used with different combinations of LED color (blue, green and red) and respective flux (E_{LED}, photons nm^{-2} 100 µs^{-1}), number of acquisitions collected (n) and average (µ) and standard deviation (±) for the optimum combination of intensity and color of the LED that fully saturates the reaction centers during the first flashlet ($R_{\sigma PII}$).

Protocols	LED colors			n	µ $R_{\sigma PII}$	± $R_{\sigma PII}$
	Blue	Green	Red			
A	0.9	0	0	821	0.47	0.0095
B	1.0	0.5	0	1083	0.57	0.0127
C	0.7	0.5	0.8	1119	0.69	0.0218
