# Supplementary material for: Contrasting phytoplankton-zooplankton distributions observed through autonomous platforms, in-situ optical sensors and discrete sampling
Source: PLoS One. 2022 Sep 6;17(9):e0273874. doi: 10.1371/journal.pone.0273874 (PMC9447933; doi:10.1371/journal.pone.0273874)

S1 Fig. Silcam data – stations 1 & 2. Scatterplot of average particle sizes (in equivalent circular diameter, µm) and concentrations (counts/L/µm) derived from the Silcam analyses binned into three depths (1-20 m, 20-40 m and 40-60 m) and for stations with deep mixed layer depth (50 m, stations 1 & 2). The shaded areas of the scatterplots in the left refer to the size spectra of particles imaged from the high (red) magnification lens that most likely represents *Tripos* spp. (100-160µm). The dashed line in the scatterplot represents the average Junge distributions. Pictures on the right side of the scatterplots in a) represent collages of particle images from the high (red box) magnification lenses.

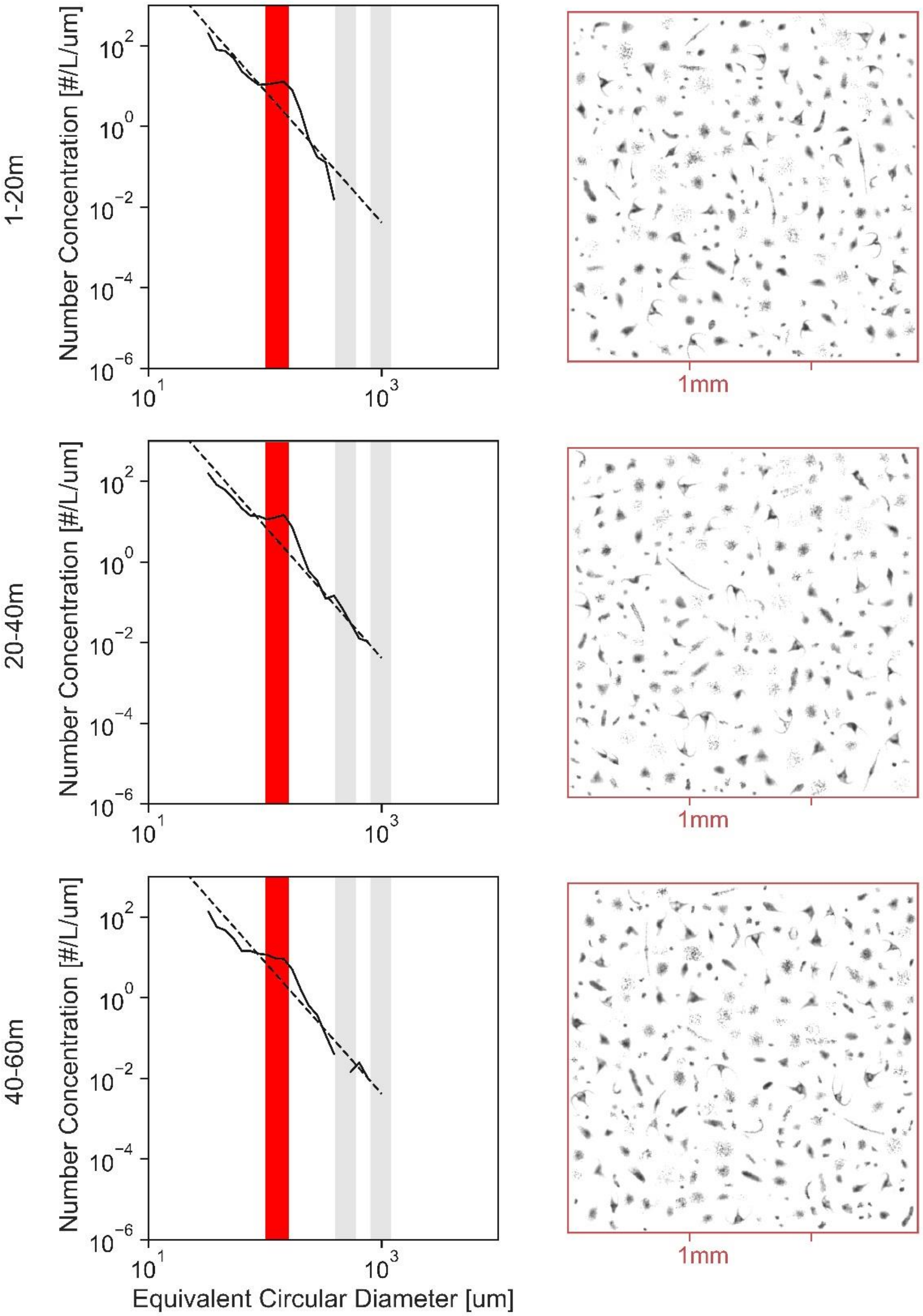

Fig S2. Scatterplot of average particle sizes (in equivalent circular diameter, µm) and concentrations (counts/L/µm) derived from the Silcam analyses binned into three depths (1-20 m, 20-40 m and 40-60 m) and for stations with shallow mixed layer depth (30 m, station 4&5). The shaded areas of the scatterplots in the left refer to the size spectra of particles imaged from the high (red) and low (blue) magnification lenses that most likely represents *Tripos* spp. (100-160µm), fecal pellet (400-6~~0~~00µm) and copepods (800-1200µm). The dashed line in the scatterplot in a) represents the average Junge distributions. Pictures on the right side of the scatterplots ~~in a)~~ represent collages of particle images from the high (red box) and low (blue box) magnification lenses.

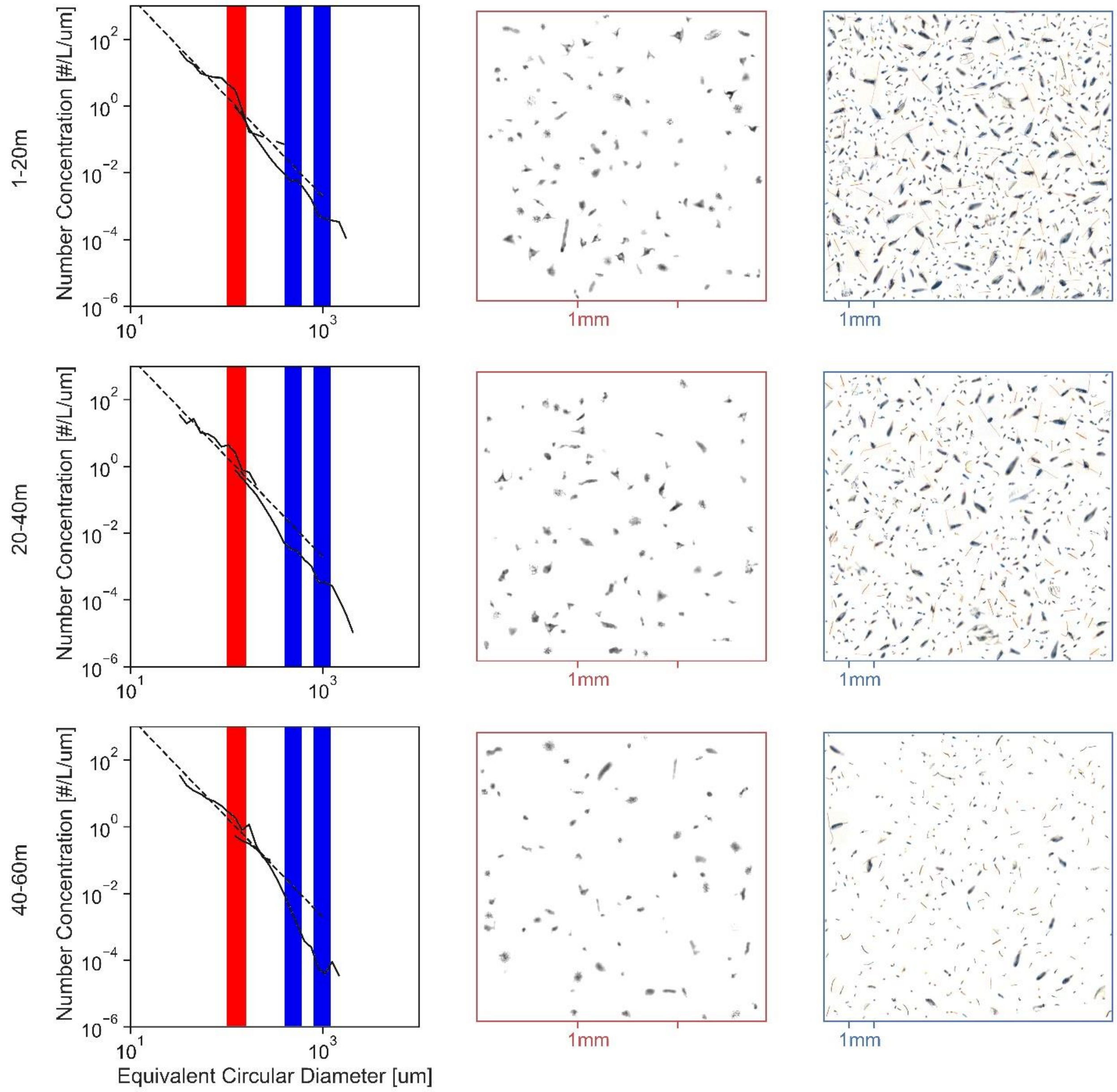

Supplement: S1 Fig — Scatterplot of average particle sizes (in equivalent circular diameter, μm) and concentrations (counts/L/μm) derived from the Silcam analyses binned into three depths (1–20 m, 20–40 m and 40–60 m) and for stations with deep mixed layer depth (50 m, stations 1&2). The shaded areas of the scatterplots in the left refer to the size spectra of particles imaged from the high (red) magnification lens that most likely represents Tripos spp. (100–160μm). The dashed line in the scatterplot in a) represents the average Junge distributions. Pictures on the right side of the scatterplots in represent collages of particle images from the high (red box) magnification lenses. (PDF) [file pone.0273874.s003.pdf]
